# Where Does HIT Fit? An Examination of the Affective Response to High-Intensity Intervals in Comparison to Continuous Moderate- and Continuous Vigorous-Intensity Exercise in the Exercise Intensity-Affect Continuum

**DOI:** 10.1371/journal.pone.0114541

**Published:** 2014-12-08

**Authors:** Mary E. Jung, Jessica E. Bourne, Jonathan P. Little

**Affiliations:** School of Health and Exercise Sciences, University of British Columbia, Okanagan, British Columbia, Canada; Pennington Biomedical Research Center, United States of America

## Abstract

Affect experienced during an exercise session is purported to predict future exercise behaviour. Compared to continuous moderate-intensity exercise (CMI), the affective response to continuous vigorous-intensity exercise (CVI) has consistently been shown to be more aversive. The affective response, and overall tolerability to high-intensity interval training (HIT), is less studied. To date, there has yet to be a comparison between HIT, CVI, and CMI. The purpose of this study was to compare the tolerability and affective responses during HIT to CVI and CMI. This study utilized a repeated measures, randomized, counter-balanced design. Forty-four participants visited the laboratory on four occasions. Baseline fitness testing was conducted to establish peak power output in Watts (Wpeak). Three subsequent visits involved a single bout of a) HIT, corresponding to 1-minute at ∼100% Wpeak and 1-minute at ∼20% Wpeak for 20 minutes, b) CMI, corresponding to ∼40% Wpeak for 40 minutes, and c) CVI, corresponding to ∼80% Wpeak for 20 minutes. The order of the sessions was randomized. Affective responses were measured before, during and after each session. Task self-efficacy, intentions, enjoyment and preference were measured after sessions. Participants reported greater enjoyment of HIT as compared to CMI and CVI, with over 50% of participants reporting a preference to engage in HIT as opposed to either CMI or CVI. HIT was considered more pleasurable than CVI after exercise, but less pleasurable than CMI at these times. Despite this participants reported being just as confident to engage in HIT as they were CMI, but less confident to engage in CVI. This study highlights the utility of HIT in inactive individuals, and suggests that it may be a viable alternative to traditionally prescribed continuous modalities of exercise for promoting self-efficacy and enjoyment of exercise.

## Introduction

There is indisputable evidence that engaging in regular exercise improves mental and physical health and reduces the risk for costly chronic diseases [1]. A single bout of exercise can lead to improved emotional well-being, including reduced anxiety [2], decreased mild depression [3], and increased energy and vigor [4] when assessed post-exercise. Despite being aware of the numerous benefits associated with exercise [5] the vast majority of Canadians (up to 85%) fail to adhere to the recommended 150 minutes of exercise each week [6]. One possible explanation provided for the high rates of inactivity is that people choose not to engage in behaviours they find aversive. Specifically, affect experienced *during* an activity has been shown to predict future engagement in that activity [7]–[9]. Placed in the context of exercise adherence, hedonic theory suggests that how one *feels during exercise*, or one's *affective response to exercise*, may predict their future exercise intentions and exercise behaviour [10].

### Exercise Intensity – Affect Relationship

Research examining the affective response during exercise in inactive and overweight adults has identified a negative relationship between exercise intensity and affect, such that as the intensity of the exercise performed increases above the ventilatory threshold, the affective response to the exercise becomes more negative [11]–[13]. Specifically, continuous bouts of vigorous-intensity exercise, such as cycling at ∼80% of VO_2_max for 30 minutes, provokes greater psychological distress [11], less enjoyment [14], [15], and higher feelings of displeasure [16] as compared to moderate-intensity cycling at ∼50% of VO_2_max. In 2003, Ekkekakis proposed the dual-mode theory as an explanation for the dramatic decline in pleasure experienced when individuals exercise above and beyond ventilatory threshold [17]. According to dual-mode theory, affect experienced during exercise is influenced, in part, by the metabolic cost associated with the intensity at which the exercise is performed. This proposed relationship has provided impetus for modification of exercise guidelines [18], [19], which recommend moderate-intensity activities, such as walking for 30 minutes 5 days per week, over vigorous-intensity activities (e.g., running for 20 minutes 3 days per week) for inactive individuals. In conflict with this recommendation is the research revealing that the most commonly cited barrier to exercise is lack of time [20]. Indeed, regardless of age, ethnicity, sex, or health status, people report that a lack of time is the primary reason for their failure to exercise on a regular basis [20]. Clearly, there is a need for innovative exercise strategies that promote health benefits with *minimal time commitment required*, and that *are not perceived as aversive*.

### Low-volume High-Intensity Interval Training (HIT): A Potential Alternative

HIT, which involves relatively brief bursts of vigorous exercise separated by periods of recovery, has been touted as a time-efficient, novel alternative to continuous exercise. Accumulating evidence indicates that HIT induces similar health-enhancing adaptations when compared to continuous exercise, despite a substantially lower time commitment [21]. While the timesaving advantage of HIT suggests it may be a promising option for increasing physical activity rates, the effectiveness of this modality of exercise is tied to the intensity at which the intervals are performed. This may be of concern, given the aforementioned research suggesting an aversive affective response to exercise performed above ventilatory threshold. As such, HIT will only be a viable alternative to more time-consuming traditional modes of continuous exercise *if* it is perceived to be enjoyable and pleasurable. Bartlett and colleagues [22] demonstrated that retrospective perceived enjoyment of an exercise bout consisting of 6×3-minute intervals at 90% VO_2_max was greater than the retrospective perceived enjoyment of running continuously at 70% VO_2_max for 50 minutes. The vast majority of literature examining the relationship between exercise intensity and in-task affect have compared vigorous-intensity *continuous* exercise to more moderate levels of *continuous* exercise. We are aware of only two recent exceptions to this. Tritter and colleagues [23] examined the moderating effects of efficacious statements (e.g., “You're doing an amazing job!”) on affect in university students performing 4×30-second *maximal* sprints. Oliveira and colleagues [24] assessed in-task affect of very high-intensity intervals in comparison to an energy-matched continuous vigorous-intensity condition, and reported that affect was lower in their HIT condition. Implications of Oliveira's findings must be taken with caution, however, as their HIT protocol was so challenging that 53% of their sample failed to complete it. Recently, a low-volume more “practical” model of HIT has been developed, which involves 10×1-minute vigorous intensity bouts separated by 1-min recovery periods [25], [26]. Inactive individuals who are overweight [27] and with type 2 diabetes [26] can complete this form of HIT and it is effective for improving their cardiometabolic health. However, the low-volume, “practical” model of HIT remains untested for its tolerability in comparison to the traditional continuous bouts of exercise. The recovery periods built into low-volume, practical HIT, and the reduced total exercise time, may make this type of vigorous exercise more pleasurable, or less aversive, than continuous vigorous-intensity exercise.

### Feasibility of HIT in Sedentary Adults

The feasibility and perceived enjoyment of HIT was tested in a pilot study of 8 inactive adults with type 2 diabetes [26]. After completing HIT involving 10×1-minute cycling intervals at ∼90% VO_2_max separated by 1-minute recovery, participants were asked about their perceived enjoyment for HIT, as well as for hypothetical bouts of 30 minutes of continuous moderate-intensity exercise and 60 minutes of continuous moderate-intensity exercise, such as brisk walking or cycling. Interestingly, sedentary adults rated HIT as more enjoyable than engaging in either 30 or 60 minutes continuous moderate-intensity exercise (*p*<.01). This preliminary investigation provided impetus for the present study, as it demonstrated that a) inactive adults are receptive to HIT; b) they are physically capable of engaging in HIT; and c) the negative relationship between affect and exercise intensity may not hold when the vigorous-intensity exercise is performed in brief bursts interspersed with periods of recovery. Additional studies have reported that HIT is feasible and attainable in overweight adults [28], [29] with favourable improvements in health outcomes.

It is not known whether the nature of HIT (short bursts of vigorous activity) will be perceived as aversive, as has been shown for continuous vigorous-intensity exercise. It is possible that the recovery periods built into HIT could decrease time commitment, decrease monotony, and provide opportunities to experience emotional responses such as a sense of pride and accomplishment after completing each interval, which in turn may alter the intensity-affect relationship and make it more enjoyable than continuous vigorous-intensity exercise. To make fair comparisons between HIT, continuous vigorous- and continuous moderate-intensity exercise, the in-task affective response between all conditions should be compared in a within-subject design. The affective response and tolerability to HIT have yet to be empirically compared to these continuous modalities. The primary objective of this study was to compare the affective response during HIT to continuous vigorous- and moderate-intensity exercise. Based on the most recent reports of post-exercise affect involving HIT, and the aforementioned potential advantages of engaging in a time-efficient workout that offers variety and surmountable challenges, it was hypothesized that in-task and post-exercise affect following HIT would be greater in comparison to continuous vigorous-intensity (CVI) exercise, but comparable to continuous moderate-intensity (CMI) exercise. The secondary objective was to assess the tolerability of HIT as compared to CVI and CMI in inactive adults by examining efficacy, intentions, enjoyment, and preference of each modality. It was hypothesized that preference, enjoyment, as well as self-efficacy and intentions for engaging in future bouts of each exercise modality would be higher in HIT as compared to CVI, but equal to CMI.

## Method

### Ethics Statement

Ethical approval was obtained from the University of British Columbia's Clinical Research Ethics Board (#H11-00759). All participants signed a written consent form.

### Participants

A total of 44 individuals were recruited from the campus community via posters and word of mouth. Eligibility criteria included 1) engaging in 2 or less bouts of aerobic exercise per week in the last 6 months and 2) establishing adequate exercise readiness using the Physical Activity Readiness Questionnaire (PAR-Q; [30]). The participants consisted of 28 women and 16 men (see Table 1 for demographic statistics). Sixty percent of participants were students, while the remainder worked either full or part time.

**Table 1:** Demographic characteristics of participants (n = 44).

Variable: Mean (SD)	Men (n = 16)	Women (n = 28)	*p* a
**Age**, years	30.94 (12.54)	35.36 (16.96)	= .33
**Height**, cm	178.97 (8.15)	165.78 (6.38)	<.01*
**Body mass**, kg	74.75 (9.45)	69.00 (16.49)	= .21
**BMI**, m/kg^−2^	23.34 (2.78)	24.92 (5.54)	= .22
**Moderate exercise bouts per week**	1.69 (1.96)	1.37 (1.39)	= .53
**Strenuous exercise bouts per week**	0.14 (.045)	0.72 (0.89)	= .03*
**W_max_(Watts)**	258.31(54.08)	164.57 (36.19)	<.01*
**VO_2_peak**, mL.kg^−1^.min^−1^	44.85 (9.39)	27.77 (6.11)	<.01*

a *p-value reflects differences in men and women based on independent samples t-tests.*

* *denotes significant differences between men and women*

### Procedure

Participants made a total of four visits to the laboratory. On the first visit participants read and signed an informed consent form, completed the PAR-Q to ensure exercise safety, and the Godin Leisure-time Exercise Questionnaire (LTEQ; [31]) was administered to ensure eligibility. In addition, participant's height and weight were taken to determine body mass index. Participants then performed a baseline fitness test to assess peak power output in Watts (Wpeak) in order to ascertain individualized training intensities. Participants performed the maximal fitness test on an electronically braked cycle ergometer (Velotron DynaFit Pro, Seattle, WA, USA) using a ramp protocol. Participants completed a 3-minute warm up, after which the test began at 50 Watts. Workload was increased by 1 Watt every 3 seconds until volitional exhaustion. Wpeak was defined as the highest workload achieved. VO_2_peak was estimated according to Storer and colleagues [32]. Participants' heart rate was recorded throughout the test (Polar FT7, Finland).

Using a randomized, counter-balanced cross-over design participants made three subsequent visits to the laboratory, spaced one week apart, to perform single bouts of 1) continuous moderate-intensity exercise (CMI); 2) continuous vigorous-intensity exercise (CVI); and 3) high-intensity interval training (HIT). All exercise trials were conducted on the cycle ergometer using a standardized warm up and cool down of 3 minutes at a self-selected light intensity (determined in the first trial and held constant for all visits). The CMI trial consisted of riding at an intensity of ∼40% Wpeak for 40 minutes (eliciting a HR of ∼69±9% HRmax). The CVI trial involved riding for 20 minutes at an intensity of ∼80% Wpeak (eliciting a HR of ∼89±7% HRmax). The HIT trial involved riding for 20 minutes, alternating between 1-minute intervals at ∼100% Wpeak (eliciting a HR of ∼90±7% HRmax) and 1 minute recovery periods at ∼20% Wpeak. CMI and CVI trials were matched for external work, while CVI and HIT were matched for time. Because one of the potential attractive features of HIT is that the exercise is low-volume in nature, it was not possible to match work across all three conditions in the current study design. Participants were informed that they could stop any of the exercise trials at any time. Following the cool-down participants were asked to remain in the laboratory for 20 minutes. Participants completed psychological measures prior to exercise, during the exercise trials, immediately post-exercise and at 20-minutes post-exercise.

### Measures

#### Exercise Intensity

Intensity of each exercise bout was monitored in two ways. The 10-point **Category-Ratio 10 Scale** (CR-10; [33], also commonly referred to as the Rating of Perceived Exertion) was used to assess participants' perceived effort during exercise. The CR-10 is a 10-point scale ranging from 0 to 10 with anchors ranging from “No exertion at all” (0) to “Maximal exertion” (10). Participants were asked to rate their exertion before (pre-exercise), immediately post and 20-minutes post-exercise. In addition participants were asked to rate their exertion at 2.5%, 7.5%, 42.5%, 47.5%, 92.5% and 97.5% of exercise completed. These time points were chosen to incorporate both interval and recovery periods during the HIT protocol and were standardized across trials. Participants' **heart rate** was recorded using Polar heart-rate monitors at 2.5%, 42.5%, and 92.5% of exercise completed.

#### Affect

The one item **Feeling Scale** (FS; [34]) was used to measure general affective valence (i.e., pleasure and displeasure). Participants are prompted at the beginning of each exercise visit with the following instructions: “While participating in exercise, it is common to experience changes in mood. Some individuals find exercise pleasurable, whereas others find it to be unpleasant. Additionally, feeling may fluctuate across time. That is, one might feel good and bad a number of times during exercise. When asked please tell me how you feel at that current moment using the scale below”. The feeling scale is scored on an 11-point bipolar scale ranging from -5 to +5. Seven anchors are provided ranging from, “Very Good” (+5) to “Very Bad” (−5). The FS was administered at pre, immediately post and 20-minutes post-exercise. To assess in-task affect, the FS was administered at 2.5%, 42.5%, and 92.5% of exercise completed.

The **Activation Deactivation Adjective Check List** (AD ACL; [35]) was used to assess participants' arousal state pre-exercise, immediately post-exercise and 20-minutes post-exercise during each exercise trial. This 20-item measure asks participants to rate a series of adjectives describing their arousal to assess the bipolar dimensions of two subscales, namely energetic arousal (e.g., active, energetic, sleepy and drowsy) and tension arousal (e.g., jittery, intense, placid and calm). Responses are scored on a 4-point rating scale using the anchors, “Definitely feel” (4), “Feel slightly” (3), “Cannot decide” (2), “Definitely do not feel” (1). Five items of the energetic arousal subscale, and three items of the tension arousal subscale, respectively; are reverse scored. Items are then summed and averaged for each subscale. This measure has been found to display strong validity and reliability [36].

#### Exercise Task Self-Efficacy

Participants' confidence in their ability to repeat the exercise they just completed was assessed at 20-minutes post-exercise using a 5-item measure. Each question included the stem, “How confident are you that you can…”. The 5-items were: 1) “perform one bout of exercise a week for the next 4 weeks that is just like the one you completed today?” 2) “Perform two bouts of exercise a week for the next 4 weeks that is just like the one you completed today?” 3) “Perform three bouts of exercise a week for the next 4 weeks that is just like the one you completed today?” 4) “Perform four bouts of exercise a week for the next 4 weeks that is just like the one you completed today?” 5) “Perform five bouts of exercise a week for the next 4 weeks that is just like the one you completed today?” Responses were scored on a scale of 0% (Not at all) to 100% (Extremely confident) in 10% increments. The specificity of the five items was created following recommendations made by Bandura and [37] and McAuley and Mihalko [38]. This measure demonstrated good internal consistency in the current study (*α'*s ≥.95).

#### Intentions

Participants' intentions to engage in the exercise just completed over the next month were assessed using a 2-item measure, 20-minutes post-exercise. Specifically, participants were asked “Please rate the extent to which you agree with the following statements 1) I intend to engage in the type of exercise I performed today at least 3 times per week during the next month” and 2) I intend to engage in the type of exercise I performed today at least 5 times per week during the next month”. Responses were scored on a 7-point rating scale with anchors ranging from “Very unlikely” (1) to Very likely (7). The two items were analyzed individually.

#### Enjoyment

Participants' enjoyment of each exercise trial was examined using a modified version of the **Physical Activity Enjoyment Scale** (PACES; [39]) 20-minutes post-exercise. This 18-item measure is scored on a 7-point bipolar scale. Example items are “it's not very refreshing/It's very refreshing” and “I would rather be doing something else/there is nothing else I would rather be doing”. The original measure was modified by deleting one of the 18 items that was irrelevant due to the time point at which we measured it (“I am very absorbed in the activity – I am not at all absorbed in the activity”). In addition, the original PACES instructions were modified from “Please rate how you feel AT THE MOMENT about the physical activity you have been doing.’ to “Think about the exercise you did today and rate your enjoyment of it”. Again, this modification was made to reflect the fact that we assessed physical activity enjoyment 20 minutes after the exercise session was completed. Items were summed to produce an overall enjoyment score out of 119. In the present study the measure was found to be internally consistent (α's ≥.96).

In addition, participants were asked two additional enjoyment questions 20-minutes post-exercise. The first, relating to participants' **enjoyment of the exercise** just completed asked; “How much did you enjoy the exercise you just completed today?”. The second question asked participants about their **anticipated enjoyment of the exercise** just completed if they were to do it again in the future. Specifically, “How enjoyable would you find engaging in this form of exercise three times per week over the next month?” Each question was scored on a 9-point rating scale with responses ranging from 1 (Not enjoyable at all) to 9 (Very enjoyable). Each question was analyzed individually.

#### Preference

At the end of the final exercise trial participants were asked to complete two questions regarding exercise preference based on participants' experiences of all exercise trials. The **first question** asked; “If it were entirely up to you, which type of exercise would you choose to do?” Three responses were available based on the three exercise modalities, these were describe as a) Endurance training at a moderate intensity level, b) Endurance training at a vigorous intensity level, or c) Sprint interval training at a vigorous intensity level. The **second question**, a three-item measure, asked participants about their fondness of the different exercise trials performed, with the instruction; “Please rank your fondness for each type of workout you performed”. Reponses were scored on a 7-point rating scale with anchors ranging from “Very much dislike” (1), “Neutral” (4) and “Extremely like” (7). Responses were analyzed individually.

### Data Analyses

Data were analyzed using SPSS Statistics (v21, 2012). Data were examined for normality and to identify potential outliers. Two repeated measures analyses of variance (RMANOVA) were conducted to ensure that participants were working at the required intensity by examining changes in exertion and heart rate between the three conditions during the exercise trials. A series of 3 (condition) by 3 (time) RMANOVA were conducted to examine differences in affect pre-, immediately post-, and 20-minutes post-exercise, as well as changes in FS during exercise (i.e., at 2.5%, 42.5% and 92.5% of exercise completed). In addition, a series of one-way RMANOVA were conducted to examine differences in self-efficacy, intentions, enjoyment, and preference following the three exercise trials. When required, pairwise comparisons were conducted using LSD corrections. Effect sizes were calculated using partial *η* ^2^ in order to examine the magnitude of the differences between the three conditions. Cohen's *d* was used to indicate the magnitude of differences between two specific groups. Data are available upon request per PLoS data policy.

## Results

### Manipulation Check

To confirm that exercise intensity was lower in CMI than in HIT and CVI, and to compare exercise intensity differences between HIT and CVI, heart rate and RPE data were analyzed across all exercise trials. The 3 (exercise trial: CMI, CVI, HIT) by 3 (time points: 2.5%, 42.5% and 92.5% of exercise completed) RMANOVA on heart rate data showed significant main effects for exercise trial, *F*(1.64, 63.99) = 381.12, *p*<.01, η_p_^2^ = .89 and time, *F*(1.35, 52.75) = 397.73, *p*<.01, η_p_^2^ = .91, as well as a significant exercise trial by time interaction, *F*(2.69, 104.85) = 41.78, *p*<.01, η_p_^2^ = .52. Average heart rate (SD) at 92.5% of exercise completed was 167.93 (14.62), 169.40 (14.24) and 127.80 (14.23) beats/min^−1^, for HIT, CVI and CMI trials respectively. Pairwise comparisons revealed that heart rate was significantly higher in the HIT and CVI trials compared to the CMI trial at 92.5% of exercise completed (*p*'s<.001). There was no difference in heart rate between the HIT and CVI trials at this time.

**Figure 1:**
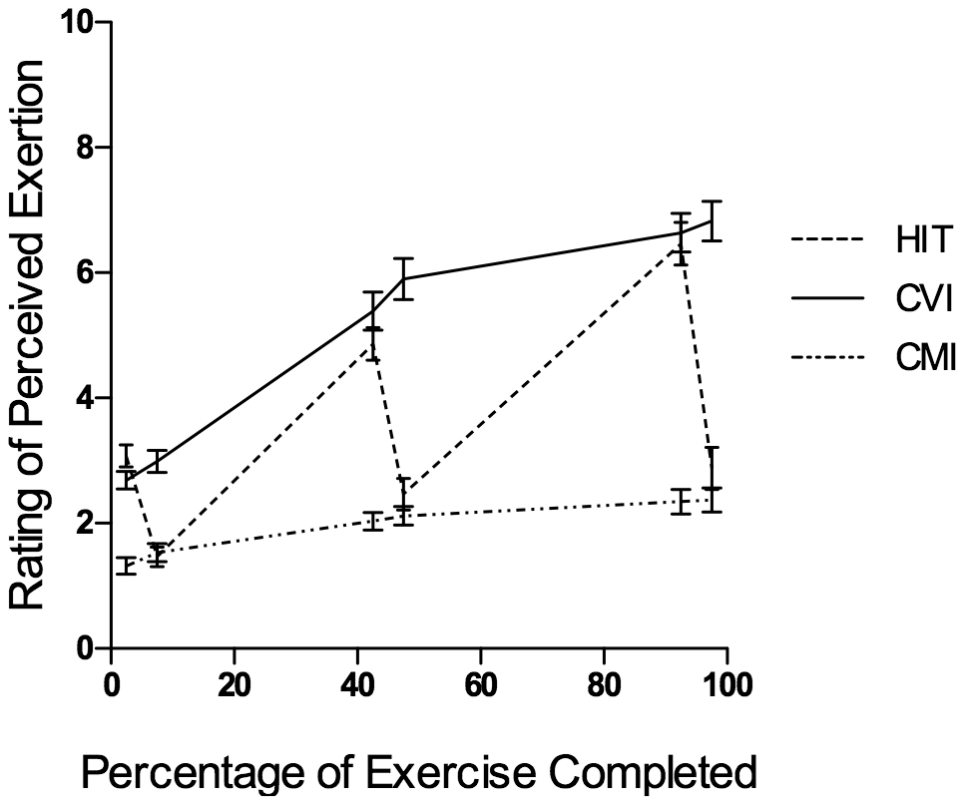
Rating of perceived exertion (M±SE) during the three exercise trials.

A 3 (exercise trial) by 6 (time points: 2.5%, 7.5%, 42.5%, 47.5%, 92.5% and 97.5% of exercise completed) RMANOVA on perceived exertion revealed significant main effects for exercise trial, *F*(2, 78) = 113.91, *p*<.01, η_p_^2^ = .75, and time, *F*(2.12, 82.60) = 110.87, *p*<.01, η_p_^2^ = .74, and a significant exercise trial by time interaction, *F*(5.23, 203.98) = 65.96, *p*<.001, η_p_^2^ = .63. Pairwise comparisons revealed that at 2.5%, 42.5% and 92.5% of exercise completed (corresponding to the 1-minute ‘on’ intervals in the HIT trial) there was a significant difference in perceived exertion between the CMI trial and both HIT and CVI conditions (*p*'s <.05) however, there was no significant difference in perceived exertion between the HIT and CVI trials at these time points (*p*'s>.05). At 7.5%, 47.5% and 97.5% of exercise completed (corresponding to the 1-minute recovery intervals in the HIT trial), comparisons revealed a significant difference in perceived exertion between the CVI trial and both the HIT and CMI trials (*p*'s <.05), but no significant difference in perceived exertion between the CMI and HIT trial at these time points (see Fig. 1).

### Affect During Exercise

**Figure 2:**
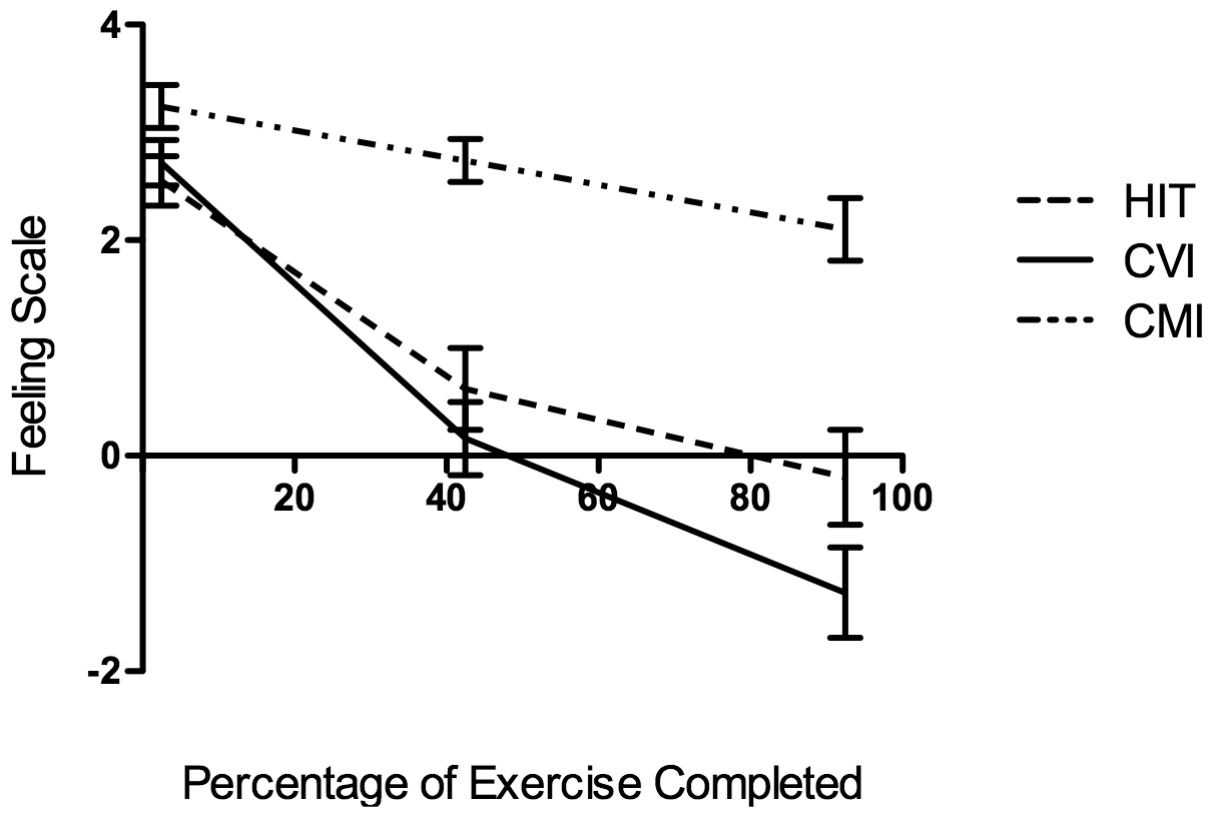
Feeling Scale responses (M±SE) during the three exercise trials.

In order to assess changes in affect during exercise, a 3 (exercise trial) by 3 (time points: 2.5%, 42.5%, and 92.5% of exercise completed) RMANOVA was conducted. Results revealed significant main effects for exercise trial, *F*(2, 78) = 44.98, *p*<.01, η_p_^2^ = .54 and time, *F*(1.25, 48.89) = 63.31, *p*<.01, η_p_^2^ = .62, and a significant exercise trial by time interaction, *F*(2.98, 116.19) = 17.16, *p*<.01, η_p_^2^ = .31. Affect decreased significantly throughout exercise in all trials (see Table 2). Affect was significantly less positive in the HIT and CVI trial than in the CMI trial at 2.5%, 42.5% and 92.5% of exercise completed (*p's*<.05). There were no significant differences in affective valence between HIT and CVI condition at 2.5% or 42.5% (*p's*>.05). However, at 92.5% of workout completed that was a significant difference between the HIT and CVI conditions (*p* = .03, *d* = 1.4). The average affect ratings at 92.5% of exercise completed (±SD) were −0.20 (±2.85), −1.27 (±2.70), and 2.10 (±1.87) for HIT, CVI and CMI trials respectively (see Fig. 2).

**Table 2:** Feeling Scales responses (mean ± SE) measured before, during and after the three exercise conditions.

Condition	Pre	2.5%	7.5%	42.5%	47.5%	92.5%	97.5%	Post Exercise	20-Mins post
**CMI**	3.15 (.21)	3.24 (.20)	3.18 (.18)	2.74 (.20)	2.65 (.21)	2.10 (.29)	2.10 (.29)	2.85 (.24)	3.61 (.18)
**HIT**	3.02 (.24)	2.55 (.23)	2.62 (.21)	0.62 (.38)	0.92 (.34)	−0.20 (.44)	0.40 (.45)	1.42 (.38)	3.29 (.20)
**CVI**	3.04 (.24)	2.72 (.21)	2.42 (.20)	0.16 (.34)	−0.53 (.39)	−1.27 (.42)	−1.54 (.44)	.84 (.38)	2.79 (.27)

### Affect Pre- to Post-exercise

To examine changes in affect pre- to post-exercise between the exercise trials, a 3 (exercise trial) by 3 (time points: pre-exercise, immediately post-exercise, 20-minutes post-exercise) RMANOVA on ratings of affective valence (FS) revealed significant main effects for exercise trial, *F*(2, 72) = 10.77, *p*<.01, η_p_^2^ = .23, and time, *F*(1.43, 51.50) = 25.60, *p*<.01, η_p_^2^ = .42, and a significant exercise trial by time interaction, *F*(3.19, 114.74) = 9.83, *p*<.01, η_p_^2^ = .21. Pairwise comparisons revealed that affect immediately post-exercise was significantly less positive in the HIT and CVI trials compared to the CMI trial (*p*'s<.01, *d*'s ≥.77) The average affect scores (SD) were 1.14 (2.50), 0.58 (2.52), and 2.74 (1.58) for HIT, CVI and CMI, respectively; immediately post-exercise. Affect improved following all trials at 20-minutes post-exercise (see Table 2). However, a significant difference remained between the CVI and CMI trials at 20-minutes post-exercise (*p* = .01, *d* = .63), while there was no significant difference between the HIT and CMI trials at 20-minutes post-exercise (*p* = .11, *d* = .28).

To examine changes in the two subscales of the AD ACL, namely; Energetic Arousal (EA) and Tension Arousal (TA), a RMANOVA was conducted. There was a significant main effect of exercise trial for EA, *F*(2,80) = 4.13, *p* = .02, η_p_^2^ = .09, and a significant main effect of exercise trial for TA, *F*(2, 80) = 13.40, *p*<.01, η_p_^2^ = .25. There was a significant main effect of time for TA, *F*(2, 80) = 25.12, *p*<.01, η_p_^2^ = .39, but not for EA. In addition, there was a significant exercise trial by time interaction for EA, *F*(3.09, 123.76) = 3.63, *p* = .02, η_p_^2^ = .08, as well as a significant exercise trial by time interaction for TA, *F*(3.30, 131.83) = 2.95, *p* = .03, η_p_^2^ = .07. From pre- to post-exercise there was a significant increase in EA following the CMI trial compared to both CVI and HIT trials, in which EA remained low. EA decreased across all trials 20-minutes post-exercise, however EA remained significantly higher following the CMI trial compared to both the HIT and CVI trials (*p*'s <.05, *d*'s ≥.35). There was a significant increase in TA following all trials immediately post-exercise, however the increase in TA was significantly greater following the HIT and CVI trials, compared to CMI trial (*p*'s <.05, *d*'s ≥.51). TA decreased 20-minutes post-exercise across all trials, however TA remained significantly lower in the CMI trial compared to HIT and CVI trials (*p*'s <.05, *d*'s ≥.79).

### Exercise Task Self-Efficacy

Differences in exercise task self-efficacy for each exercise trial were assessed using a one-way RMANOVA. There was a significant main effect between exercise trials, *F*(2, 82) = 6.58, *p*<.01, η_p_^2^ = .14. Specifically, participants felt significantly more confident that they could perform HIT and CMI as compared to CVI (*p*'s <.05, *d*'s ≥.34). No significant differences were seen between HIT and CMI (*p* = .28, *d* = .14).

### Exercise Intentions

Intentions to perform the different exercise trials either three times per week or five times per week were assessed using two, one-way RMANOVA. Analyses revealed a significant difference in intentions to exercise 3 times per week between the trials, *F*(2, 80) = 6.41, *p*<.01, η_p_^2^ = .14, and in intentions to exercise 5 time per week between the trials, *F*(2, 80) = 6.08, *p* = <.01, η_p_^2^ = .13. In both cases, participants were significantly more likely to set intentions to engage in HIT or CMI as compared to CVI (*p*'s <.05, *d*'s ≥.48). There were no significant differences in intentions to engage in HIT compared to CMI (*p*'s>.50, *d*'s ≤.25).

### Exercise Enjoyment

**Figure 3:**
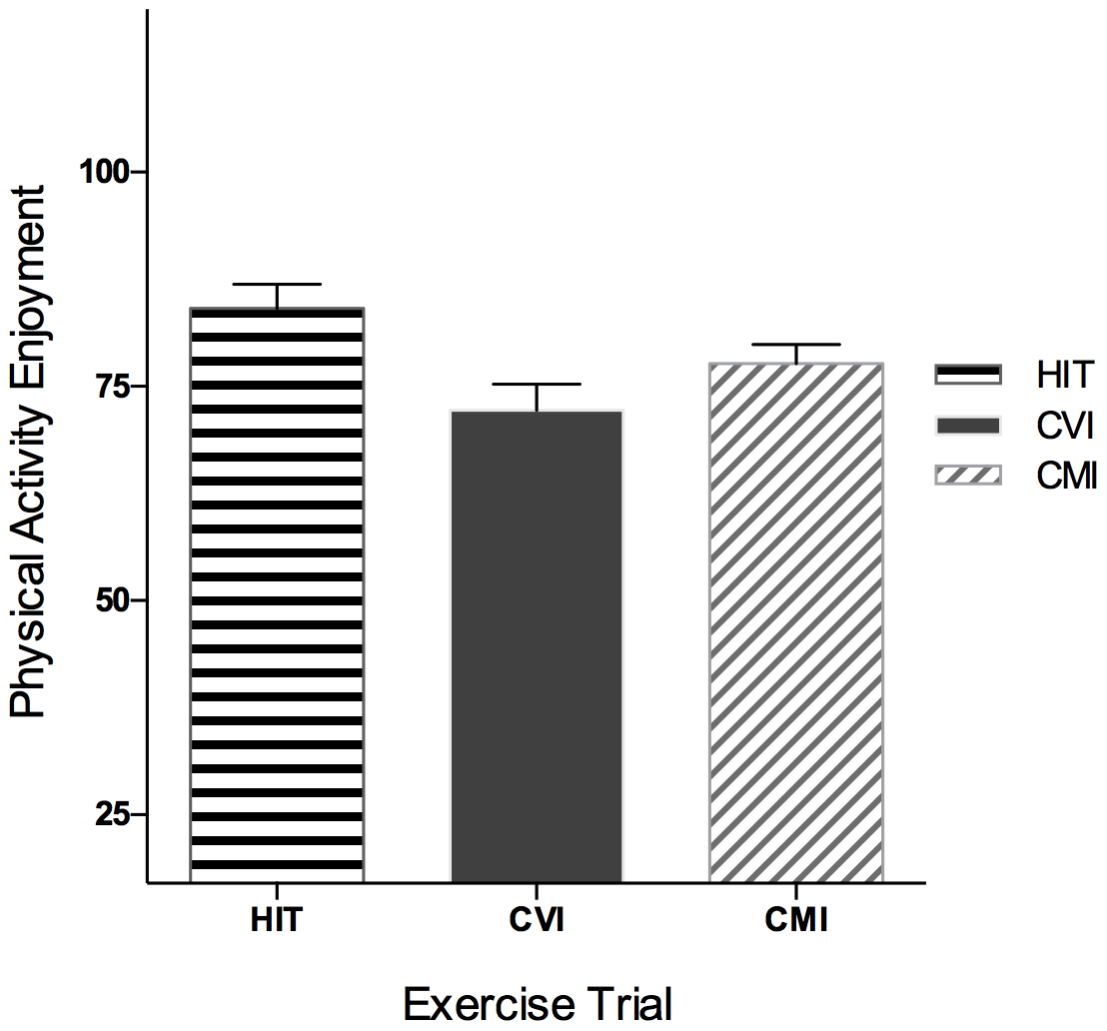
Enjoyment (M±SE) of the three exercise trials.

To examine differences in enjoyment of the three exercise trials using the **Physical Activity Enjoyment Scale** [39], a one-way RMANOVA on physical activity enjoyment revealed a significant main effect of exercise trial, *F*(2, 80) = 5.77, *p*<.01, η_p_^2^ = .13. Pairwise comparisons revealed that the HIT trial was rated as significantly more enjoyable than CVI (*p* = .01, *d* = .64). HIT was not statistically more enjoyable than CMI at *p* = .08, however examination of the means and calculation of effect size revealed a medium sized effect (*d* = .43) favoring HIT. Furthermore, CMI was not considered more enjoyable than CVI (*p* = .11 *d* =  0.32; see Fig. 3).

To examine **enjoyment of exercise modality** a one-way RMANOVA revealed similar results, *F*(2, 80) = 8.21, *p* = .01, η_p_^2^ = .17, with HIT and CMI trials being rated as significantly more enjoyable than the CVI trial (*p*'s <.01, *d*'s ≥.62) and no significant differences in enjoyment between the HIT and CMI trials (*p* = .74, *d* = .07).

To examine differences in **anticipated exercise modality enjoyment** between exercise trials a one-way RMANOVA was conducted. This analysis revealed a significant difference in anticipated exercise modality enjoyment between the trials, *F*(2, 80) = 8.92, *p*<.01, η_p_^2^ = .18. Pairwise comparisons revealed that participants were significantly more likely to anticipate enjoying HIT or CMI exercise than CVI (*p*'s <.01, *d*'s ≥.64). There was no significant difference in anticipated enjoyment between HIT and CMI exercise (*p* = .60, *d* = .12).

### Exercise Preference

To assess participants' **preference of exercise modality**, a one-way RMANOVA was conducted. The RMANOVA revealed a significant difference in exercise preference, *F*(1.58, 68.09) = 8.61, *p* = .01, η_p_^2^ = .18. Specifically, pairwise comparisons revealed a significantly greater preference for both HIT and CMI compared to CVI (*p*'s <.05, *d*'s ≥.55). Interestingly, the results suggested a preference for HIT over CMI with results nearing significance (*p* = .07, *d* = .51). Twenty-four participants reported HIT as their preferred exercise modality of choice, 13 participants reported preference CMI, and 4 reported preference for CVI.

To assess participants' fondness of the exercise trials, a one-way RMANOVA was conducted. Results yielded a significant difference between trials, *F*(2, 82) = 11.91, *p* = .01, η_p_^2^ = .23. Again, participants were significantly more fond of HIT and CMI than they were of CVI (*p*'s <.01, *d*'s ≥.97). There was no difference in fondness of HIT and CMI (*p* = .59, *d* = .14).

## Discussion

Longer, moderate-intensity bouts of exercise have been advocated over shorter, higher-intensity sessions on the premise of a negative relationship between affect and exercise intensity once one reaches and surpasses ventilatory threshold. There is little data on the affective response to high-intensity exercise when performed in an intermittent fashion, and none that we are aware of that has empirically tested the affective response to HIT to more traditional continuous forms of moderate- and vigorous-intensity exercise. To our knowledge, this is the first study of its kind to examine affective responses to three different exercise intensities before, during and after exercise. The secondary purpose was to assess the tolerability of HIT as compared to CMI and CVI.

The findings of the current study provide some support for our hypothesis that there would be more pleasant affective responses to the HIT trial as compared to the CVI trial. Specifically, HIT was considered more pleasurable than CVI when assessed using the activation-deactivation adjective checklist, and there was a tendency for more pleasant in-task affect as assessed by the feeling scale nearing the end of the workout (92.5% completed) in HIT as compared to CVI at *p* = .03 and an effect size of 1.4. This suggests that there is an underlying effect deserving of further investigation. Taken together, these findings are intriguing given that participants were working at a higher intensity during the HIT trial (100% vs. 80% of Wpeak in CVI). The affective responses to HIT and CVI observed in the present study counter findings with a previous study examining the affective response to HIT [24]. Oliveira and colleagues [24] recently compared affect between HIT and energy-matched continuous vigorous-intensity exercise and found that in-task affect was more positive in their continuous vigorous-intensity condition. These equivocal findings may be a result of methodological differences. Eight of their 15 participants could not complete their HIT protocol due to difficulty level, whereas in the current study the purpose was to assess a low-volume practical model of HIT. We tested a 1∶1 ratio of work to rest, lasting 60 seconds each, whereas Oliveira and colleagues [24] utilized 2-minute work intervals with less than 60 seconds of recovery between each. The fact that their participants reported considerably less pleasure than our participants in HIT conditions is therefore not unexpected. We chose our low-volume HIT protocol based on past research showing that this exercise strategy is attainable and effective in inactive individuals who are overweight/obese [40] and with type 2 diabetes [26], [41].

In contrast to our hypothesis that affect scores would be similar between HIT and CMI, HIT was reported to be less pleasurable than CMI both during and after exercise in our sample. The greater decrease in positive affect seen during and after HIT as compared to CMI is likely due to the greater intensity at which individuals are working, as predicted by the exercise intensity – affect relationship [17]. However, there appears to be a unique aspect to HIT, such that a) affect responses during exercise are not as negative as those seen in CVI; and b) there is an apparent positive rebound effect 20-minutes post HIT that is not seen in CVI. It is important to note that in all exercise trials, in-task affect significantly decreased, such that in all trials participants reported less positive affect over time. Interestingly, during the low intensity (recovery) intervals incorporated into HIT, participants perceived that they were working at a substantially lower intensity than during the high-intensity intervals (i.e., recovering and having time to recharge) despite their heart rates remaining elevated.

Despite differences in affect between the HIT and CMI trials, participants reported feeling just as confident in their ability to perform HIT as they did CMI, while confidence was substantially lower for completing CVI exercise. This finding is in line with our hypothesis and demonstrates that HIT consists of specific characteristics conducive with bolstering self-efficacy that are not seen when working vigorously in a continuous manner. It is possible that the intermittent nature of the high intensity intervals enables participants to experience a number of successive positive accomplishments that are not available during continuous vigorous exercise. Specifically, due to the ‘on-off’ nature of HIT participants may believe they are capable of pushing harder for a perceivably short period of time (i.e., 1-minute) before getting a break, while during CVI participants are asked to push hard continuously for 20 minutes. As such, HIT breaks down the exercise sessions into short, surmountable bursts, potentially allowing for multiple successful experiences, which in turn could serve to increase self-efficacy beliefs. Given that individuals are inherently drawn to engage in behaviours that they feel confident they can carry out [37], it is not surprising that participants in the current study reported being more likely to set future intentions to engage in HIT or CMI but that future intentions for CVI were low, as predicted.

In regards to the tolerability of HIT, findings were consistent with predictions. Specifically, participants reported greater enjoyment of HIT and CMI compared to CVI as measured through PACES, enjoyment of the specific exercise modality, and anticipated exercise modality enjoyment. As far as we are aware this is the first study to examine exercise enjoyment across HIT, CMI and CVI collectively. Interestingly, PACES results are in line with Barlett and colleagues (2011), who found participants enjoyed HIT more than continuous moderate-intensity type exercise, although the differences between HIT and CMI in our study did not reach statistical significance (*p* = .08). Building on this, the current PACES data revealed that participants did not rate CMI exercise as any more enjoyable than CVI exercise.

Finally, and most strikingly, participants displayed a greater preference for HIT over both CVI and CMI exercise despite less pleasant affective responses in HIT as compared to CMI. Specifically, 24 individuals reported a preference to engage in HIT, as compared to only 4 and 13 individuals who preferred to engage in CVI and CMI, respectively. It is possible that this preference for HIT stems from individuals' perceived confidence to perform HIT, coupled with greater enjoyment of the exercise and the reduced time commitment required.

Considered globally, these findings support the evidence that CVI can lead to negative psychological responses [11], [15], [16]. However, HIT does not appear to elicit such prominent negative psychological responses as those seen during and after CVI. It is plausible that the intermittent nature of HIT evokes a series of breaks from negative affective responses. Over and above decreasing monotony of continuous exercise, intervals may serve to cause a “rebound effect” with affect, such that during recovery intervals participants feel considerably more pleasure. The work intervals may be serving to repeatedly bolster confidence within a single exercise session, as well as increase enjoyment through the continual perceived switch between ‘on-off’ work. Consequently, participants have the ability to push themselves out of their ‘comfort’ zone for a known, and perceivably manageable, period of time with the knowledge of an approaching period of recovery before performing the same behaviour again. This enables participants to tackle each interval individually rather than the constant strain required during CVI exercise.

As mentioned previously, differences in study findings may be a result of methodological differences. For example, in their HIT protocol Bartlett and colleagues [22] utilized a 3-minute high intensity interval at 90% of VO_2_peak, with a 3-minute rest interval at 50% of VO_2_peak, while their CMI condition ran for 50 minutes at 70% of VO_2_peak. Oliveira and colleagues [24] employed high intensity intervals at 100% of VO_2_peak lasting 2 minutes, with recovery at 0% VO_2_peak lasting ∼57 seconds – less than half of the time of their work intervals. Previous research has demonstrated the utility of the repeated 1-minute on∶off (∼100% VO_2_peak;∼20% VO_2_peak) HIT protocol we used in our study in achieving positive physiological adaptations [40]–[42]. Thus, the HIT protocol employed in the present study appears to be highly enjoyed, was preferred by a majority of inactive adults, and may have greater applicability compared to these previous HIT studies.

The findings of the current study suggest that HIT may be a viable alternative to continuous moderate-intensity exercise prescriptions for samples similar to the inactive individuals studied in this study, however individual differences on affective responses or preferences may exist and future research needs to explore long-term adherence of HIT in relation to CMI. These study findings support dual-mode theory in that the more vigorous-intensity exercise (CVI and HIT) resulted in less positive affect, but suggest that there are marked differences between continuous vigorous-intensity exercise and intermittent vigorous exercise. As such, not all exercise performed at or above ventilatory threshold should be treated equally. In addition, tolerability and perceptions of various forms of HIT should be explored in other populations (e.g., individuals with chronic disease) in order to establish potential differences in affective responses and tolerability of intensity level.

Overall, this study provides preliminary findings demonstrating that HIT leads to less displeasure compared to CVI, and less pleasure compared to CMI in a sample of inactive adults. Despite this, participants report HIT as more enjoyable and the preferred exercise modality compared to CVI and comparable to CMI. As such, this study highlights the potential utility of HIT for use within the general population and its comparable, and in some cases beneficial, impact on various psychological constructs in comparison to traditionally prescribed continuous-moderate intensity exercise.

## References

[1] Warburton DE, Nicol CW, Bredin S (2006) Health benefits of physical activity: the evidence. Canadian Medical Association Journal 174:801–809. 16534088 10.1503/cmaj.051351 PMC1402378

[2] Tate AK, Petruzzello SJ (1995) Varying the intensity of acute exercise: implications for changes in affect. The Journal of sports medicine and physical fitness 35:295–302. 8776078

[3] Morgan WP (1994) Psychological components of effort sense. Medicine & Science in Sports & Exercise 26:1071–1077. 7808238

[4] O'Connor PJ, Puetz TW (2005) Chronic physical activity and feelings of energy and fatigue. Medicine & Science in Sports & Exercise 37:299–305. 15692327 10.1249/01.mss.0000152802.89770.cf

[5] Warburton D, Charlesworth S, Ivey A, Nettlefold L, Bredin S (2010) A systematic review of the evidence for Canada's Physical Activity Guidelines for Adults. International Journal of Behavioral Nutrition and Physical Activity 7:39. 20459783 10.1186/1479-5868-7-39 PMC3583166

[6] Colley RC, Garriguet D, Janssen I, Craig CL, Clarke J, et al (2011) Physical activity of Canadian adults: accelerometer results from the 2007 to 2009 Canadian Health Measures Survey. Component of Statistics Canada Health Reports 22:1–8. 21510585

[7] Emmons RA, Diener E (1986) A goal-affect analysis of everyday situational choices. Journal of Research in Personality 20:309–326.

[8] Williams DM, Dunsiger S, Ciccolo JT, Lewis BA, Albrecht AE, et al (2008) Acute Affective Response to a Moderate-intensity Exercise Stimulus Predicts Physical Activity Participation 6 and 12 Months Later. Psychology of Sport and Exercise 9:231–245. 18496608 10.1016/j.psychsport.2007.04.002 PMC2390920

[9] Williams DM, Dunsiger S, Jennings EG, Marcus BH (2012) Does Affective Valence During and Immediately Following a 10-Min Walk Predict Concurrent and Future Physical Activity? Annals of Behavioral Medicine 44:43–51. 22532005 10.1007/s12160-012-9362-9 PMC5718347

[10] Ekkekakis P (2009) Let Them Roam Free? Sports Medicine 39:857–888. 19757863 10.2165/11315210-000000000-00000

[11] Blanchard CM, Rodgers WM, Spence JC, Courneya KS (2001) Feeling state responses to acute exercise of high and low intensity. Journal of science and medicine in sport/Sports Medicine Australia 4:30–38. 10.1016/s1440-2440(01)80005-0 11339491

[12] Ekkekakis P, Pretruzzello SJ (2002) Analysis of the affect measurement conundrum in exercise psychology: IV. A conceptual case for the affect circumplex. Psychology of Sport and Exercise 2:35–63.

[13] Parfitt G, Hughes S (2009) The Exercise Intensity–Affect Relationship: Evidence and Implications for Exercise Behavior. Journal of Exercise Science & Fitness 7:S34–S41.

[14] Brewer BW, Manos TM, McDevitt AV, Cornelius AE, Van Raalte JL (2000) The effect of adding lower intensity work on perceived aversiveness of exercise. Journal of Sport & Exercise Psychology 22:119–130.

[15] Kilpatrick M, Hebert E, Bartholomew J, Hollander D, Stromberg D (2003) Effect of Exertional Trend during Cycle Ergometry on Postexercise Affect. Research Quarterly for Exercise and Sport 74:353–359. 14510303 10.1080/02701367.2003.10609103

[16] Hall EE, Ekkekakis P, Petruzzello SJ (2002) The affective beneficence of vigorous exercise revisited. British Journal of Health Psychology 7:47–66. 14596717 10.1348/135910702169358

[17] Ekkekakis P (2003) Pleasure and displeasure from the body: Perspectives from exercise. Cognition & Emotion 17:213–239. 10.1080/02699930302292 29715726

[18] Physical Activity Guidelines Advisory Committee (2008) Physical Activity Guidelines Advisory Committee Report. Washington, DC, U. S.: Department of Health and Human Services.

[19] Garber CE, Blissmer B, Deschenes MR, Franklin BA, Lamonte MJ, et al. (2011) Quantity and Quality of Exercise for Developing and Maintaining Cardiorespiratory, Musculoskeletal, and Neuromotor Fitness in Apparently Healthy Adults: Guidance for Prescribing Exercise. Medicine & Science in Sports & Exercise 43: 1334–1359 1310.1249/MSS.1330b1013e318213fefb. 10.1249/MSS.0b013e318213fefb 21694556

[20] Trost SG, Owen N, Bauman AE, Sallis JF, Brown W (2002) Correlates of adults' participation in physical activity: review and update. Medicine & Science in Sports & Exercise 34:1996–2001. 12471307 10.1097/00005768-200212000-00020

[21] Gibala MJ, McGee SL (2008) Metabolic adaptations to short-term high-intensity interval training: a little pain for a lot of gain? Exercise and Sport Science Review 36:58–63. 10.1097/JES.0b013e318168ec1f 18362686

[22] Bartlett JD, Close GL, MacLaren DPM, Gregson W, Drust B, et al (2011) High-intensity interval running is perceived to be more enjoyable than moderate-intensity continuous exercise: Implications for exercise adherence. Journal of Sports Sciences 29:547–553. 21360405 10.1080/02640414.2010.545427

[23] Tritter A, Fitzgeorge L, Cramp AG, Valiulis P, Prapavessis H (2013) Self-efficacy and affect responses to Sprint Interval Training. Psychology of Sport and Exercise 14:886–890.

[24] Oliveira BRR, Slama FA, Deslandes AC, Furtado ES, Santos TM (2013) Continuous and High-Intensity Interval Training: Which Promotes Higher Pleasure? PLOS one 8:e79965. 24302993 10.1371/journal.pone.0079965 PMC3841165

[25] Little JP, Safdar A, Wilkin GP, Tarnopolsky MA, Gibala MJ (2010) A practical model of low-volume high-intensity interval training induces mitochondrial biogenesis in human skeletal muscle: potential mechanisms. The Journal of Physiology 588:1011–1022. 20100740 10.1113/jphysiol.2009.181743 PMC2849965

[26] Little JP, Gillen JB, Percival ME, Safdar A, Tarnopolsky MA, et al (2011) Low-volume high-intensity interval training reduces hyperglycemia and increases muscle mitochondrial capacity in patients with type 2 diabetes. Journal of Applied Physiology 111:1554–1560. 21868679 10.1152/japplphysiol.00921.2011

[27] Hood M, Little JP, Tarnopolsky MA, Frank M, Gibala MJ (2011) Low-Volume Interval Training Improves Muscle Oxidative Capacity in Sedentary Adults. Medicine & Science in Sports & Exercise 43:1849–1856. 21448086 10.1249/MSS.0b013e3182199834

[28] Trapp EG, Chisholm DJ, Freund J, Boutcher SH (2008) The effects of high-intensity intermittent exercise training on fat loss and fasting insulin levels of young women. Int J Obes 32:684–691. 10.1038/sj.ijo.0803781 18197184

[29] Heydari M, Freund J, Boutcher SH (2012) The Effect of High-Intensity Intermittent Exercise on Body Composition of Overweight Young Males. Journal of Obesity 2012:8. 10.1155/2012/480467 PMC3375095 22720138

[30] CSEP (2002) Physical Activity Readiness Questionnaire In: Physiology CSoEeditor.

[31] Godin G, Shepard RJ (1997) Godin Leisure-Time Exercise Questionnaire. Medicine and Science in Sports and Exercise 29:S36–S38.

[32] Storer TW, Davis JA, Caiozzo VJ (1990) Accurate prediction of VO2max in cycle ergometry. Medicine & Science in Sports & Exercise 25:704–712. 10.1249/00005768-199010000-00024 2233211

[33] Borg G (1998) Borg's Perceived exertion and pain scales; Kinetics Heditor.

[34] Hardy CJ, Rejeski WJ (1989) Not what, but how one feels: the measurement of affect during exercise. Journal of Sport and Exercise Psychology 11:304–317.

[35] Thayer RE (1989) The Biopsychology of Mood and Arousal. New York: Oxford University Press.

[36] Ekkekakis P, Hall EE, Petruzzello SJ (2005) Evaluation of the circumplex structure of the Activation Deactivation Adjective Check List before and after a short walk. Psychology of Sport and Exercise 6:83–101.

[37] Bandura A (1997) Self-efficacy: The exercise of control. New York: W. H. Freeman & Co.

[38] McAuley E, Mihalko SL (1998) Measuring exercise-related self-efficacy. In: Duda JLeditor. Advances in sport and exercise psychology measurement USA: Fitness Information, Inc. pp. 371–381.

[39] Kendzierski D, DeCarlo KJ (1991) Physical Activity Enjoyment Scale: Two validation studies. Journal of Sport & Exercise Psychology 13:50–64.

[40] Little JP, Jung ME, Wright AE, Wright W, Manders RJF (In press) Effects of high-intensity interval exercise versus continuous-moderate intensity exercise on postprandial glycemic control assessed by continuous glucose monitoring in obese adults. Applied Physiology, Nutrition, and Metabolism. 10.1139/apnm-2013-0512 24773254

[41] Gillen JB, Little JP, Punthakee Z, Tarnopolsky MA, Riddell MC, et al (2012) Acute high-intensity interval exercise reduces the postprandial glucose response and prevalence of hyperglycaemia in patients with type 2 diabetes. Diabetes, Obesity and Metabolism 14:575–577. 10.1111/j.1463-1326.2012.01564.x 22268455

[42] Little JP, Gillen JB, Percival M, Safdar A, Tarnopolsky MA, et al (2011) Low-volume high-intensity interval training reduces hyperglycemia and increases muscle mitochondrial capacity in patients with type 2 diabetes. Journal of Applied Physiology 111:1554–1560. 21868679 10.1152/japplphysiol.00921.2011

